# Modeling of Compressive Strength for Self-Consolidating High-Strength Concrete Incorporating Palm Oil Fuel Ash

**DOI:** 10.3390/ma9050396

**Published:** 2016-05-20

**Authors:** Md. Safiuddin, Sudharshan N. Raman, Md. Abdus Salam, Mohd. Zamin Jumaat

**Affiliations:** 1 Angelo Del Zotto School of Construction Management, George Brown College, 146 Kendal Avenue, Toronto, ON M5T 2T9, Canada; 2 Department of Architecture, Universiti Kebangsaan Malaysia, UKM Bangi 43600, Selangor, Malaysia; 3 Department of Civil Engineering, Dhaka University of Engineering & Technology, Gazipur-1700, Dhaka 1213, Bangladesh; masalam@duet.ac.bd; 4 Department of Civil Engineering, University of Malaya, Kuala Lumpur 50603, Malaysia; zamin@um.edu.my

**Keywords:** artificial neural network (ANN), compressive strength, modeling, palm oil fuel ash (POFA), self-consolidating high-strength concrete (SCHSC)

## Abstract

Modeling is a very useful method for the performance prediction of concrete. Most of the models available in literature are related to the compressive strength because it is a major mechanical property used in concrete design. Many attempts were taken to develop suitable mathematical models for the prediction of compressive strength of different concretes, but not for self-consolidating high-strength concrete (SCHSC) containing palm oil fuel ash (POFA). The present study has used artificial neural networks (ANN) to predict the compressive strength of SCHSC incorporating POFA. The ANN model has been developed and validated in this research using the mix proportioning and experimental strength data of 20 different SCHSC mixes. Seventy percent (70%) of the data were used to carry out the training of the ANN model. The remaining 30% of the data were used for testing the model. The training of the ANN model was stopped when the root mean square error (RMSE) and the percentage of good patterns was 0.001 and ≈100%, respectively. The predicted compressive strength values obtained from the trained ANN model were much closer to the experimental values of compressive strength. The coefficient of determination (*R*^2^) for the relationship between the predicted and experimental compressive strengths was 0.9486, which shows the higher degree of accuracy of the network pattern. Furthermore, the predicted compressive strength was found very close to the experimental compressive strength during the testing process of the ANN model. The absolute and percentage relative errors in the testing process were significantly low with a mean value of 1.74 MPa and 3.13%, respectively, which indicated that the compressive strength of SCHSC including POFA can be efficiently predicted by the ANN.

## 1. Introduction

Tropical countries such as Malaysia, Indonesia and Thailand are palm oil producing nations; among them, Malaysia is the world’s leading manufacturer and exporter of palm oil and palm oil products with around 41% of the total world supply [1]. There are about more than 200 palm oil mills operating in Malaysia [2]. From these palm oil mills, a significant amount of waste is produced in the form of nutshells, fibers, and empty bunches after the extraction of oil from the fresh fruit bunches [3,4,5]. This waste is used in generating electrical energy for milling operations and domestic or estate use in the palm oil mills [6,7]. After combustion, approximately 5% ash by weight of waste is produced [8,9]; this ash is referred to as palm oil fuel ash (POFA). Usually, POFA is dumped in an open field near the palm oil mills, thus creating environmental pollution and a health hazard [3,8,10]. To find a sustainable solution, several studies have been conducted to examine the viability of using POFA in different types of concrete. The research outcomes confirmed that finely ground POFA can be used successfully as a supplementary cementing material (SCM) for producing various types of concrete. This has been substantiated by the findings of Safiuddin *et al.* [9], Karim *et al.* [10], Ranjbar *et al.* [11] and Kabir *et al.* [12]. However, no comprehensive research was conducted to explore the potential of POFA for the production of self-consolidating high-strength concrete (SCHSC). The research on the development of SCHSC incorporating POFA has been carried out by the authors and the outcome shows that the compressive strength of SCHSC is improved in the presence of POFA with up to 20% weight replacement of cement.

The 28-day compressive strength is an important criterion in selecting a concrete for a particular application. Therefore, the prediction of compressive strength has been an active area of concrete research. Different tools along with artificial neural networks (ANN) have been used to model the compressive strength of concrete. ANN can be defined as a data processing system consisting of a large number of elements (artificial neurons) in an architecture [13]. This technique has been applied for civil/structural engineering applications for more than two decades. Adeli [14] conducted a review of the literature (published between 1989 and 2000) on the use of neural networks in various civil/structural engineering applications. The first published study thoroughly discussing the application of ANN in civil/structural engineering was probably by Adeli and Yeh [15]. Subsequently, this technique has been adopted by many researchers for use as a problem-solving tool in various civil/structural engineering applications [16,17,18,19,20,21,22,23,24,25]. Flood and Kartam [16,17] provided an in-depth discussion on the versatility of ANN as a problem-solving instrument, and demonstrated how it can be leveraged within the civil engineering domain.

In recent years, the ANN technique has been used by many researchers for different structural engineering applications [18]. The applications of this technique in structural engineering include the work by Dahou *et al.* [19], where the ANN was used as a modeling tool for the characteristics of bond between concrete and ribbed steel bars. Garzon-Roca *et al.* [20] utilized the ANN, based on the findings of 96 laboratory tests, to model the compressive strength of a masonry structure which was constructed with clay bricks and cement mortar. In another related study, Garzon-Roca *et al.* [21] adopted the ANN to predict the maximum axial load that can be sustained by the masonry structure. Aguilar *et al.* [22] modeled the in-plane shear strength of the reinforced masonry walls using a large experimental database and ANN. Asteris *et al.* [23] adopted the ANN to predict the fundamental period of infilled reinforced concrete frame structures. Most recently, researchers have also utilized the ANN to model the masonry failure surface under biaxial compressive stress [24,25].

In the field of concrete technology, the ANN technique has also been used by many researchers to predict the compressive strength and other properties of different concretes. Yeh [26], Kasperkiewicz *et al.* [27], Lai and Serra [28], and Lee [29] applied the ANN for predicting the compressive strength of conventional and high-performance concretes. Öztaş *et al.* [30] and Hakim *et al.* [31] developed ANN models for the prediction of compressive strength of high-strength concrete. Dias and Pooliyadda [32] used the back propagation technique for the prediction of strength and slump of conventional and high-strength concretes. Bai *et al.* [33] developed an ANN model for the workability of concrete incorporating fly ash and metakaolin. Topçu and Sarıdemir [34] predicted the compressive strength of fly ash concrete by using the ANN technique. Meanwhile, Adhikary and Mutsuyoshi [35] adopted the ANN technique to develop a model for predicting the ultimate shear strength of steel fiber–reinforced concrete (SFRC) beams, whereas Kumar and Barai [36] presented an ANN model to compute the ultimate shear strength of vertically loaded SFRC corbels without shear reinforcements. Parichatprecha and Nimityongskul [37] used the ANN technique to analyze the durability characteristics of high-performance concrete by considering the influence of water and cement contents, the water-to-binder (W/B) ratio, and the partial replacement of cement with fly ash and silica fume.

The literature review, as discussed above, reveals that no research has been undertaken on the use of ANN to predict the compressive strength of SCHSC including POFA as an SCM. The present study is aimed at building a model to predict the compressive strength of SCHSC incorporating POFA using ANN. For the purpose of constructing this model, the mix proportioning and experimental strength data of 20 different SCHSC mixes were used. In training and testing of the ANN model, cement (C), POFA, water (W), coarse aggregate (CA), fine aggregate (FA), high-range water reducer (HRWR), and viscosity modifying admixture (VMA) were entered as the inputs while the compressive strength values were used as the output. The model was trained with 70% of the data and then the remaining 30% of the data were used for testing of the model.

## 2. Materials and Methodology

### 2.1. Constituent Materials

Crushed granite stone (coarse aggregate), mining sand (fine aggregate), ordinary (ASTM Type I) Portland cement (main cementing material), POFA (supplementary cementing material), normal tap water, and a polycarboxylate-based HRWR were used in this study to produce SCHSC mixes. In addition, VMA was used when needed to improve the segregation resistance of concrete. POFA was used for substituting 0%–30% ordinary Portland cement (OPC) by weight. POFA and OPC together acted as the binder (B) for aggregates. The major physical properties of the constituent materials are given in Table 1.

### 2.2. Mix Proportions of Concretes

In the present study, 20 SCHSC mixes were prepared in total with different W/B ratios and POFA contents. The W/B ratios of 0.25, 0.30, 0.35, and 0.40 were chosen to produce the SCHSC mixes. In these concrete mixes, OPC was partially replaced with 0%, 10%, 20%, 25%, and 30% POFA by weight. The ACI 211.4R-08 [38] guideline was used to estimate the amount of mix water. The amount of cement was calculated based on the selected W/B ratios and estimated water content. The optimum fine aggregate to total aggregate (FA/TA) ratio was determined based on the maximum bulk density of the aggregate blends which was 0.50 for all SCHSC mixes. The HRWR dosages were fixed from the trial mixes to ensure that the concretes had self-consolidation capacity with a slump flow in the range of 600–800 mm. When needed, the manufacture’s recommended dosage of VMA was used to improve the segregation resistance of the concrete mixes. The SCHSC mixes were designated based on their W/B ratio and POFA content. The details of concrete mix proportions and designations are given in Table 2.

### 2.3. Preparation and Testing of Concretes

The fresh SCHSCs were prepared according to the procedure described in Safiuddin [39]. Immediately after the completion of mixing, the slump flow of the fresh concretes was determined to examine their self-consolidation capacity according to ASTM C 1611/C 1611M-09a [40]. Then the fresh SCHSC was cast to prepare the cylinder specimens of Ø100 × 200 mm to be used for the compressive strength test of concrete. Upon completion of casting, the specimens were left undisturbed and covered with a plastic sheet and wet burlap to avoid the evaporation of mix water. The specimens were de-molded, marked, and transferred to the curing tank for wet curing at the age of 24 ± 4 h. The wet curing was continued until the day of testing. For modeling, the compressive strength of the concretes was determined at the age of 28 days by testing triplicate Ø100 × 200 mm cylinder specimens in accordance with ASTM C 39/C 39M [41].

## 3. ANN for Predicting Compressive Strength of SCHSC

**Figure 1:**
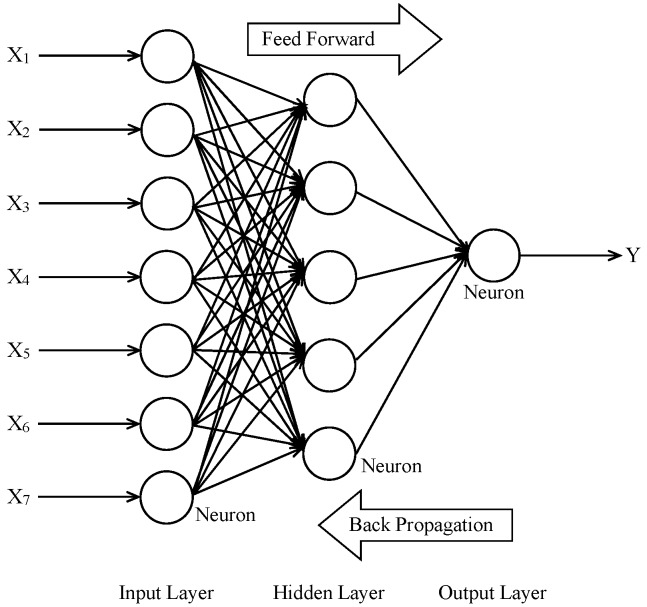
Architecture of a typical multilayer feed-forward neural network.

ANN is a non-linear modeling tool that can solve many engineering problems without using any mathematical equations. It works by relating the input and output data. The basic strategy for developing a model with the ANN technique is to train the data obtained from the experiments [18]. The trained ANN system is capable of predicting the results from other experiments [42]. The processing elements of the ANN consist of many simple computational parameters similar to the neurons in the brain [26]. The typical ANN has one input layer, one or more hidden layers, and one output layer as shown in Figure 1. Each layer of an ANN consists of many cells or neurons, which are interconnected via weights. The input data enter the ANN neurons, comprising the input layer. The input layer transmits the input data to the hidden layer(s) without any mathematical calculation [43]. The hidden layers have hidden processing units that are composed of weights, the sum function, and the activation function. The weights are values that affect an input data set on the process element. The sum function calculates the total effect of inputs and weights on the process element. The activation function processes the net input gathered from the sum function and determines the output [34].

The ANN model to predict the compressive strength of SCHSC incorporating POFA was developed using a multilayered feed-forward neural network with a back propagation algorithm (refer to Figure 1). In a feed-forward ANN, the neurons or cells are arranged in layers and the neurons of one layer are interconnected with the neurons of the consecutive layer [44]. Moreover, the back propagation algorithm is mostly used in ANNs. In this algorithm, the network error propagates back from the output layer to the input layer and the weights are adjusted to minimize the network error [43]. The accuracy of the ANN model is judged by the root mean square error (RMSE), momentum rate (it relates with the degree of good patterns), and coefficient of determination (*R*^2^). The absolute error as well as the relative error can also be used to justify the accuracy of the ANN model.

## 4. Test Results and Modeling

### 4.1. Compressive Strength of Concretes

SCHSC is significantly different from conventional concrete with respect to the constituent materials and mix parameters (W/B ratio, cement content, water content, FA/TA ratio, *etc*.). It requires some special ingredients such as HRWR and VMA. Also, SCHSC should be designed based on a W/B ratio ≤0.40. The compressive strength of SCHSC mixes in the present study was controlled by proportioning OPC, POFA, coarse and fine aggregates, water, HRWR and VMA properly for the W/B ratios ranging from 0.25 to 0.40. The average compressive strength values of SCHSCs obtained at 28 days are given in Table 3. It is evident from Table 3 that the 28-day compressive strength of different SCHSC mixes ranged from 52.3 to 74.2 MPa. This compressive strength range satisfied the strength requirement for high-strength concrete [45]. The highest compressive strength was achieved for the SCHSC with the W/B ratio of 0.25% and 20% POFA. In contrast, the SCHSC produced with the W/B ratio of 0.40% and 30% POFA provided the lowest compressive strength. These results indicate that 20% POFA optimized the gain in compressive strength. The optimum FA/TA ratio and the micro-filling ability of POFA produced dense concrete with minimum voids [46]. In addition, the pozzolanic reaction of POFA contributed to the increase in the compressive strength of the concrete by improving the pore structure of the bulk binder paste and strengthening the interfacial bond between the binder paste and aggregate [47].

### 4.2. ANN Model Development for Compressive Strength of SCHSC and Analysis

**Figure 2:**
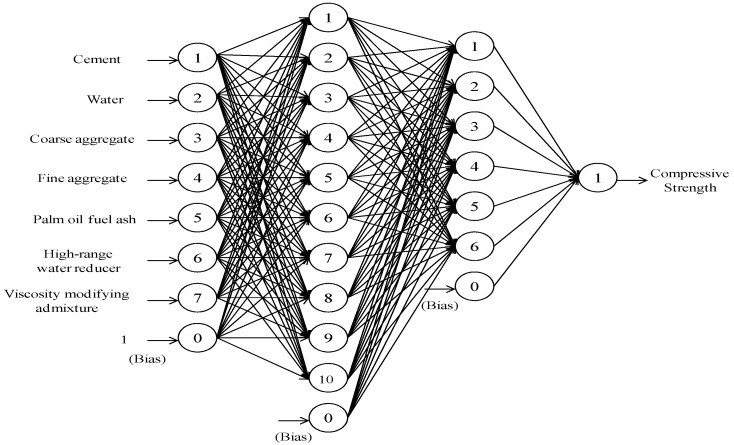
Final architecture of the ANN model.

The ANN technique has been used to predict the compressive strength of SCHSC incorporating POFA. In the present study, seven neurons in the input layer and one neuron in the output layer were used to develop the ANN model, as shown in Figure 2. Seven ingredients were used in the current study to produce various SCHSCs; these are cement, water, coarse aggregate, fine aggregate, POFA, HRWR, and VMA. The compressive strength of SCHSC is influenced by the aforementioned seven ingredients. Therefore, seven input nodes were chosen while developing the ANN model. Having the above-mentioned seven input nodes, the target node was the compressive strength of the SCHSC. In simulating the ANN, the MATLAB software was used to write the computer program code.

The mix proportioning and strength data of 20 SCHSC mixes were used in the model. These data were arbitrarily divided into 70% for the training phase and 30% for the testing phase of the ANN model. The input and output variables and the details of the mix proportioning and compressive strength values used in training the ANN model are given in Table 4 and Table 5, respectively. After being received, the input data were transmitted to the hidden layers without any processing. The neurons of the hidden layers then processed the transmitted data and extracted the useful features to reconstruct the mapping from the input domain. There were two hidden layers in the ANN model. Basically, the choice of hidden layers influences the quality of the training pattern in the ANN [30]. The maximum limit of the hidden layers was five in this ANN program. It was observed that the RMSE and the momentum rate were not at acceptable levels when one or more than two hidden layers were used. In contrast, the quality of the training pattern was much better when two hidden layers were used. This is why two hidden layers have been chosen in the present study to develop the ANN model. After processing the data in the two hidden layers, the output neuron predicted the compressive strength of SCHSC. The tansig transfer function for the hidden layers and the pureline transfer function for the output layer have been used in this study.

#### 4.2.1. Training of the ANN Model

The ANN model was trained using 70% of the mix proportioning and strength data of the SCHSC. The maximum number of epochs (iterations) was 5000. While training the network, a RMSE close to zero, a percentage of good patterns close to 100%, and a *R*^2^ (coefficient of determination) value close to 1 were sought for better accuracy of the model. The training was stopped when the RMSE reached a value ≤0.001. The stable ANN with a low RMSE was achieved by controlling the learning rate, which ranges from “0.0” to “1.0”. The learning rate should be chosen as high as possible to allow the fast learning without leading to oscillations [26]. However, when a very high learning rate is added to the network, the RMSE becomes high; this is because the network becomes unable to learn or store the knowledge when the learning rate is very high [31]. Therefore, a relatively low learning rate was used in the present study to minimize the RMSE. Also, the percentage of good patterns in the ANN depends on the momentum rate, which varies from “0.0” to “1.0”. In general, a high momentum rate is desirable to increase the speed of training of the network [48]. Hence, a relatively high momentum rate was used in this study to achieve the percentage of good patterns close to 100% in the trained ANN.

**Figure 3:**
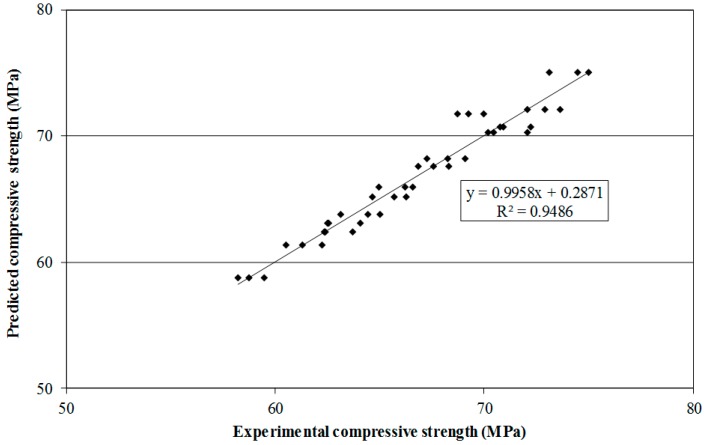
Comparison between experimental and predicted compressive strengths in training phase.

The predicted compressive strength obtained from the trained ANN model was compared with the experimental compressive strength by plotting the data in Figure 3. It is obvious from Figure 3 that the predicted and experimental compressive strengths were strongly correlated with a linear relationship. The training data are much closer to the experimental data with the coefficient of determination of 0.9486; thus, the training process seems very accurate. It has also been found that the percentage relative error of the training process ranges from 0.00041% to 4.39% (calculated based on the predicted and experimental compressive strength values) which shows the higher degree of accuracy of the network pattern. The outcome of the training process reveals that the compressive strength of SCHSC containing POFA can be predicted using the four-layer feed-forward back propagation neural network. A similar finding was reported by Topçu and Sarıdemir [43] who showed that the ANN might be used to predict the compressive strength of fly ash concrete.

#### 4.2.2. Testing of the ANN Model

After the training process, the ANN model was tested for validation using another set of data. The experimental data used for testing the model were not used in the training process. The mix proportioning and strength data used in the testing or validation process are presented in Table 6. The experimental compressive strength data and the predicted compressive strength values obtained from the ANN model during the testing process are presented in Table 7.

In the testing phase, the absolute error and the relative error were calculated to determine the accuracy of the validation process. The absolute error was computed by subtracting the experimental compressive strength from the predicted compressive strength. The relative error was determined by dividing the absolute error with the experimental compressive strength and then expressing it in percentage form. The values of the absolute and relative errors are given in Table 7. The absolute error varied from 0.1 MPa to 3.5 MPa with the mean value of 1.74 MPa. Also, it is evident from Table 7 that the relative error in the testing process ranged from 0.17% to 6.74% with a mean value of 3.13%. Both the mean absolute error and the mean relative error indicate that the errors were significantly low. According to Hakim *et al.* [31], the aforementioned absolute and relative errors are acceptable for the validation of the ANN model. Hence, it is concluded that the compressive strength of SCHSC incorporating POFA can be predicted with the ANN with a very low error rate.

## 5. Conclusions

ANN can be a viable computational tool for the prediction of concrete compressive strength. This study revealed the feasibility of using ANN for capturing non-linear interactions between various mix ingredients of SCHSC. Based on the results of the present study related to modeling of the compressive strength for SCHSC incorporating POFA, the following conclusions are drawn:

- The average 28-day compressive strength of different SCHSCs was in the range of 52.3–74.2 MPa which fulfilled the requirement for high-strength concrete. The compressive strength of the concrete was influenced by its mix parameters (particularly the W/B ratio) and mix proportions (particularly the cement and POFA contents). The highest compressive strength was obtained for the concrete produced with a W/B ratio of 0.25 and 20% POFA.
- A model has been developed using an ANN to predict the 28-day compressive strength of SCHSC containing POFA. The key information regarding the mix ingredients of concrete was used in choosing the neurons for the ANN. A multilayered feed-forward neural network with a back propagation algorithm was used to develop the model. While developing the ANN model, 70% of the mix proportioning and strength data were used in the training phase whereas 30% of the data were used in testing phase.
- The predicted compressive strength values obtained from the trained ANN model were much closer to the experimental values of compressive strength which shows the higher degree of accuracy of the created network pattern.
- The derived ANN model predicted the compressive strength of SCHSC containing POFA with a minimum error. The mean absolute error as well as the mean relative error was significantly low, as observed during the testing process of the model.
- The overall findings of the present study indicate that the compressive strength of SCHSC incorporating POFA can be efficiently predicted by using an ANN.

## Figures and Tables

**Table 1:** Physical properties of concrete constituent materials.

Coarse Aggregate (CA):	
Relative density (specific gravity)	2.62
Maximum size (mm)	19
Absorption (wt %)	0.55
Moisture content (wt %)	0.27
**Fine Aggregate (FA):**	
Relative density (specific gravity)	2.69
Maximum size (mm)	4.75
Absorption (wt %)	1.32
Moisture content (wt %)	0.31
**Ordinary Portland Cement (OPC):**	
Relative density (specific gravity)	3.16
Median particle size, d_50_ (µm)	14.6
Fraction passing 45-µm sieve (wt %)	91.5
Specific surface area, Blaine (m^2^/kg)	351
Specific surface area, BET (m^2^/kg)	3046
**Palm Oil Fuel Ash (POFA):**	
Relative density (specific gravity)	2.48
Median particle size, d_50_ (µm)	9.5
Fraction passing 45 µm sieve (wt %)	95
Specific surface area, Blaine (m^2^/kg)	775
Specific surface area, BET (m^2^/kg)	4103
**High-Range Water Reducer (HRWR):**	
Relative density (specific gravity)	1.05
Solid content (wt %)	30
**Viscosity Modifying Admixture (VMA):**	
Relative density (specific gravity)	1.01
Solid content (wt %)	20

**Table 2:** Details of mix proportions for different SCHSC mixes.

Concrete Designation	W/B Ratio	CA	FA	OPC	POFA		Water	HRWR	VMA
		(kg/m^3^)	(kg/m^3^)	(kg/m^3^)	(% B)	(kg/m^3^)	(kg/m^3^)	(kg/m^3^)	(kg/m^3^)
C25P0	0.25	767.1	762.2	705.9	0	0	178.3	12.10	0
C25P10	0.25	759.0	754.24	635.3	10	70.6	177.9	12.40	0
C25P20	0.25	750.9	746.1	564.7	20	141.2	177.9	12.60	1.76
C25P25	0.25	746.8	742.1	529.4	25	176.5	177.0	13.49	3.53
C25P30	0.25	742.8	738.1	494.1	30	211.8	176.5	14.11	5.29
C30P0	0.30	816.3	811.1	588.2	0	0	181.7	8.40	0
C30P10	0.30	809.6	804.4	529.4	10	58.8	181.3	8.82	0
C30P20	0.30	802.8	797.7	470.6	20	117.6	180.3	10.08	0
C30P25	0.30	799.4	794.4	441.2	25	147.1	179.9	10.50	1.47
C30P30	0.30	796.1	791.0	411.8	30	176.5	179.7	10.78	2.94
C35P0	0.35	851.5	846.1	504.2	0	0	184.2	5.70	0
C35P10	0.35	845.7	840.3	453.8	10	50.4	183.9	5.90	0
C35P20	0.35	839.9	834.6	403.4	20	100.8	183.7	6.05	0
C35P25	0.35	837.0	831.7	378.2	25	126.1	182.9	7.20	0
C35P30	0.35	834.1	828.8	352.9	30	151.3	182.6	7.56	0
C40P0	0.40	877.8	872.3	441.2	0	0	185.6	4.20	0
C40P10	0.40	872.8	867.3	397.1	10	44.1	185.4	4.41	0
C40P20	0.40	886.8	862.2	352.9	20	88.2	184.9	5.04	0
C40P25	0.40	865.2	859.7	330.9	25	110.3	184.8	5.15	1.10
C40P30	0.40	862.7	857.2	308.8	30	132.4	184.2	5.88	2.21

**Table 3:** Compressive strength of different SCHSC mixes at the age of 28 days.

Concrete Designation	W/B Ratio	POFA (% B)	Average Compressive Strength (MPa)
C25P0	0.25	0	70.9
C25P10	0.25	10	72.9
C25P20	0.25	20	74.2
C25P25	0.25	25	68.2
C25P30	0.25	30	65.9
C30P0	0.30	0	67.6
C30P10	0.30	10	69.3
C30P20	0.30	20	71.3
C30P25	0.30	25	65.5
C30P30	0.30	30	63.1
C35P0	0.35	0	61.3
C35P10	0.35	10	62.8
C35P20	0.35	20	64.2
C35P25	0.35	25	58.8
C35P30	0.35	30	57.7
C40P0	0.40	0	56.2
C40P10	0.40	10	57.9
C40P20	0.40	20	58.2
C40P25	0.40	25	54.1
C40P30	0.40	30	52.3

**Table 4:** Input and output variables used in the ANN model.

Input/Output Variables	Ranges of Data	
	Minimum	Maximum
**Inputs:**		
Coarse aggregate (kg/m^3^)	742.8	877.8
Fine aggregate (kg/m^3^)	738.1	872.3
Ordinary portland cement (kg/m^3^)	308.8	705.9
Palm oil fuel ash (kg/m^3^)	0	211.8
Water (kg/m^3^)	176.5	185.6
High-range water reducer (kg/m^3^)	4.20	14.11
Viscosity modifying admixture (kg/m^3^)	0	5.29
**Output:**		
Compressive strength (MPa)	52.3	74.2

**Table 5:** Details of the mix proportions and compressive strength values of concretes used in the training process of the ANN model.

Concrete Type	W/B Ratio	Constituent Materials (kg/m^3^)							Compressive Strength (MPa)
		CA	FA	OPC	POFA	W	HRWR	VMA	
C25P0	0.25	767.1	762.2	705.9	0.0	178.3	12.10	0.00	70.2
	0.25	767.1	762.2	705.9	0.0	178.3	12.10	0.00	70.4
	0.25	767.1	762.2	705.9	0.0	178.3	12.10	0.00	72.1
C25P10	0.25	759.0	754.2	635.3	70.6	177.9	12.40	0.00	73.6
	0.25	759.0	754.2	635.3	70.6	177.9	12.40	0.00	72.9
	0.25	759.0	754.2	635.3	70.6	177.9	12.40	0.00	72.1
C25P20	0.25	750.9	746.1	564.7	141.2	177.7	12.60	1.76	73.1
	0.25	750.9	746.1	564.7	141.2	177.7	12.60	1.76	74.5
	0.25	750.9	746.1	564.7	141.2	177.7	12.60	1.76	75.0
C25P25	0.25	746.8	742.1	529.4	176.5	177.0	13.49	3.53	68.3
	0.25	746.8	742.1	529.4	176.5	177.0	13.49	3.53	67.3
	0.25	746.8	742.1	529.4	176.5	177.0	13.49	3.53	69.1
C25P30	0.25	742.8	738.1	494.1	211.8	176.5	14.11	5.29	65.0
	0.25	742.8	738.1	494.1	211.8	176.5	14.11	5.29	66.2
	0.25	742.8	738.1	494.1	211.8	176.5	14.11	5.29	66.6
C30P0	0.30	816.3	811.1	588.2	0.0	181.7	8.40	0.00	66.8
	0.30	816.3	811.1	588.2	0.0	181.7	8.40	0.00	67.6
	0.30	816.3	811.1	588.2	0.0	181.7	8.40	0.00	68.3
C30P10	0.30	809.6	804.4	529.4	58.8	181.3	8.82	0.00	69.3
	0.30	809.6	804.4	529.4	58.8	181.3	8.82	0.00	70.0
	0.30	809.6	804.4	529.4	58.8	181.3	8.82	0.00	68.7
C30P20	0.30	802.8	797.7	470.6	117.6	180.3	10.08	0.00	72.2
	0.30	802.8	797.7	470.6	117.6	180.3	10.08	0.00	70.8
	0.30	802.8	797.7	470.6	117.6	180.3	10.08	0.00	70.9
C30P25	0.30	799.4	794.4	441.2	147.1	179.9	10.50	1.47	65.7
	0.30	799.4	794.4	441.2	147.1	179.9	10.50	1.47	64.6
	0.30	799.4	794.4	441.2	147.1	179.9	10.50	1.47	66.3
C30P30	0.30	796.1	791.0	411.8	176.5	179.7	10.78	2.94	64.1
	0.30	796.1	791.0	411.8	176.5	179.7	10.78	2.94	62.5
	0.30	796.1	791.0	411.8	176.5	179.7	10.78	2.94	62.6
C35P0	0.35	851.5	846.1	504.2	0.0	184.2	5.70	0.00	60.5
	0.35	851.5	846.1	504.2	0.0	184.2	5.70	0.00	62.2
	0.35	851.5	846.1	504.2	0.0	184.2	5.70	0.00	61.3
C35P10	0.35	845.7	840.3	453.8	50.4	183.9	5.90	0.00	62.4
	0.35	845.7	840.3	453.8	50.4	183.9	5.90	0.00	63.7
	0.35	845.7	840.3	453.8	50.4	183.9	5.90	0.00	62.4
C35P20	0.35	839.9	834.6	403.4	100.8	183.7	6.05	0.00	64.4
	0.35	839.9	834.6	403.4	100.8	183.7	6.05	0.00	63.1
	0.35	839.9	834.6	403.4	100.8	183.7	6.05	0.00	65.0
C35P25	0.35	837.0	831.7	378.2	126.1	182.9	7.20	0.00	59.5
	0.35	837.0	831.7	378.2	126.1	182.9	7.20	0.00	58.2
	0.35	837.0	831.7	378.2	126.1	182.9	7.20	0.00	58.7

**Table 6:** Details of the mix proportions and compressive strength values of concretes used in the testing process of ANN model.

Concrete Type	W/B Ratio	Constituent Materials (kg/m^3^)						
		CA	FA	OPC	POFA	W	HRWR	VMA
C35P30	0.35	834.1	828.8	352.9	151.3	182.6	7.56	0.00
	0.35	834.1	828.8	352.9	151.3	182.6	7.56	0.00
	0.35	834.1	828.8	352.9	151.3	182.6	7.56	0.00
C40P0	0.40	877.8	872.3	441.2	0.0	185.6	4.20	0.00
	0.40	877.8	872.3	441.2	0.0	185.6	4.20	0.00
	0.40	877.8	872.3	441.2	0.0	185.6	4.20	0.00
C40P10	0.40	872.8	867.3	397.1	44.1	185.4	4.41	0.00
	0.40	872.8	867.3	397.1	44.1	185.4	4.41	0.00
	0.40	872.8	867.3	397.1	44.1	185.4	4.41	0.00
C40P20	0.40	886.8	862.2	352.9	88.2	184.9	5.04	0.00
	0.40	886.8	862.2	352.9	88.2	184.9	5.04	0.00
	0.40	886.8	862.2	352.9	88.2	184.9	5.04	0.00
C40P25	0.40	865.2	859.7	330.9	110.3	184.8	5.15	1.10
	0.40	865.2	859.7	330.9	110.3	184.8	5.15	1.10
	0.40	865.2	859.7	330.9	110.3	184.8	5.15	1.10
C40P30	0.40	862.7	857.2	308.8	132.4	184.2	5.88	2.21
	0.40	862.7	857.2	308.8	132.4	184.2	5.88	2.21
	0.40	862.7	857.2	308.8	132.4	184.2	5.88	2.21

**Table 7:** Comparison of the predicted and experimental compressive strengths in the testing phase.

Experimental Compressive Strength (MPa)	Predicted Compressive Strength from ANN Model (MPa)	Absolute Error (MPa)	Relative Error (%)
57.9	58.2	0.3	0.52
58.2	58.3	0.1	0.17
56.9	58.2	1.3	2.28
55.1	55.7	0.6	1.09
56.5	55.8	0.7	1.24
57.0	55.8	1.2	2.10
58.9	55.9	3.0	5.09
57.0	55.9	1.1	1.93
57.9	55.9	2.0	3.45
58.6	55.7	2.9	4.95
57.6	55.7	1.9	3.30
58.4	55.7	2.7	4.62
54.0	55.5	1.5	2.78
53.9	55.5	1.6	2.97
54.4	55.5	1.1	2.02
51.9	55.4	3.5	6.74
52.0	55.4	3.4	6.54
53.0	55.4	2.4	4.53
		Mean: 1.74	Mean: 3.13

## Abbreviations

The following abbreviations are used in this manuscript:

ANN	Artificial neural network
C	Cement
CA	Coarse aggregate
FA	Fine aggregate
HRWR	High-range water reducer
SCHSC	Self-consolidating high-strength concrete
SCM	Supplementary cementing material
POFA	Palm oil fuel ash
VMA	Viscosity modifying admixture
W	Water
W/B	Water-to-binder ratio
